# Truffle Brûlés Have an Impact on the Diversity of Soil Bacterial Communities

**DOI:** 10.1371/journal.pone.0061945

**Published:** 2013-04-30

**Authors:** Antonietta Mello, Guo-Chun Ding, Yvette M. Piceno, Chiara Napoli, Lauren M. Tom, Todd Z. DeSantis, Gary L. Andersen, Kornelia Smalla, Paola Bonfante

**Affiliations:** 1 Plant Protection Institute, Turin UOS, CNR, Italy; 2 Julius Kühn-Institut, Messeweg, Braunschweig, Germany; 3 Lawrence Berkeley National Laboratory, Berkeley, California, United States of America; 4 Department of Life Science and Systems Biology, Turin, Italy; 5 Second Genome Inc., San Bruno, California, United States of America; University of Waterloo, Canada

## Abstract

**Background:**

The development of *Tuber melanosporum* mycorrhizal symbiosis is associated with the production of an area devoid of vegetation (commonly referred to by the French word ‘brûlé’) around the symbiotic plants and where the fruiting bodies of *T. melanosporum* are usually collected. The extent of the ecological impact of such an area is still being discovered. While the relationship between *T. melanosporum* and the other fungi present in the brûlé has been assessed, no data are available on the relationship between this fungus and the bacteria inhabiting the brûlé.

**Methodology/Principal Findings:**

We used DGGE and DNA microarrays of 16S rRNA gene fragments to compare the bacterial and archaeal communities inside and outside of truffle brûlés. Soil samples were collected in 2008 from four productive *T. melanosporum/Quercus pubescens* truffle-grounds located in Cahors, France, showing characteristic truffle brûlé. All the samples were analyzed by DGGE and one truffle-ground was analyzed also using phylogenetic microarrays. DGGE profiles showed differences in the bacterial community composition, and the microarrays revealed a few differences in relative richness between the brûlé interior and exterior zones, as well as differences in the relative abundance of several taxa.

**Conclusions/Significance:**

The different signal intensities we have measured for members of bacteria and archaea inside *versus* outside the brûlé are the first demonstration, to our knowledge, that not only fungal communities, but also other microorganisms are affected by *T. melanosporum*. *Firmicutes* (e.g., *Bacillus*), several genera of *Actinobacteria*, and a few *Cyanobacteria* had greater representation inside the brûlé compared with outside, whereas *Pseudomonas* and several genera within the class *Flavobacteriaceae* had higher relative abundances outside the brûlé. The findings from this study may contribute to future searches for microbial bio-indicators of brûlés.

## Introduction

*Tuber melanosporum* Vittadini is an ectomycorrhizal fungus that grows in symbiosis with several oak species and hazelnut trees in France, Italy, and the Iberian Peninsula [1]. It also has been introduced successfully from these Mediterranean areas to New Zealand, Australia, Israel, and North America [2]. The worldwide culinary demand for *T. melanosporum* is due to its unique aroma and flavor. The sequencing of its genome has represented a breakthrough that has led to expanded knowledge of the biology of this fungus [3]. *T. melanosporum,* at 125 megabases, is one of the largest fungal genomes sequenced to date. Transposable elements, accounting for 58% of the genome, are responsible for this expansion. The most important outcome from this genome survey concerns the life cycle of *T. melanosporum*, as the analysis of genes involved in the mating process has demonstrated that *T. melanosporum* is an obligate out-crossing species. Understanding of the propagation mode of this fungus likely will provide new applications for its cultivation [3].

The development of *T. melanosporum* is associated with the production of an area around the symbiotic plants that looks burned (commonly referred to by the French word ‘brûlé’), where the fruiting bodies of *T. melanosporum,* the black truffles, usually are collected. The brûlé is an area devoid of vegetation, usually circular, and because of this, is easily recognizable. Many studies have demonstrated that specific truffle volatiles can be perceived by plants and potentially inhibit the growth of some [4]. One study hypothesized parasitism of the *Tuber* spp. on the non-host herbaceous plants [5], whereas another study hypothesized that *Tuber* ectomycorrhizas may out-compete plants for nutrients and water [6]. The full ecological effects of such a niche, as well as the dynamics of the microorganisms living there, are yet to be discovered. Previous work on the composition of fungal communities inside and outside of *T. melanosporum* brûlés revealed clear differences between the fungal communities [7]. A lower fungal biodiversity inside the brûlé was suggested by two biodiversity indices. Molecular cloning revealed that *T. melanosporum* was the dominant fungus within the brûlé where Basidiomycota ectomycorrhizal fungi decreased. These decreases indicated a competitive effect of *T. melanosporum* on the other ectomycorrhizal fungi. This fungal dominance was confirmed by 454-pyrosequencing data of the same soils [8], and suggests truffles have an efficient strategy of ensuring their survival by spreading their metabolites, regarded as having allelopathic effects on the herbaceous plants and the microorganisms of the rhizosphere [9].

Changes in the vegetation or fungal communities are important for soil bacteria, so one would predict there would be some effect of the brûlé on bacterial communities. Would there be a measurable impact on the structure of the bacteria and archaea living in the brûlé? Would microbe-mediated mechanisms affect or be affected by *T. melanosporum* fruiting body production? It is known that truffle fruiting bodies enclose a broad bacterial community, according to their hypogeous status. Well-defined bacterial communities were found in completely immature fruiting bodies, suggesting a role in the first steps of fruiting body formation in the soil [10]. Given that ectomycorrhizal fungi interact with soil communities to establish a multi-trophic ectomycorrhizal complex [11], [12], a causal relationship between the productive niches and the resident microbial communities is a potential scenario. Our aim was to compare the bacterial communities between brûlé interior and exterior zones to learn about potential effects of *T. melanosporum* growth and metabolites on such communities.

Two complimentary molecular methods were chosen to perform the comparison: DGGE and DNA microarrays. DGGE has been used extensively to profile prokaryotic community composition from numerous samples [13]. DNA microarrays have been applied because of their high sensitivity and breadth of coverage. In fact, they are able to show differences in relative abundance for sequences present in amplicon pools over approximately five orders of magnitude [14]. PhyloChip arrays are comprised of multiple oligonucleotide probes for all known prokaryotic taxa for breadth of coverage, and the pairing of a mismatch probe for every perfectly matched probe is designed to minimize the effect of non-specific hybridization [15]. In order to study the bacterial and archaeal communities inside and outside of the brûlé, soil samples were collected in 2008 from four productive *T. melanosporum*/*Quercus pubescens* truffle-grounds located in Cahors, France, all having hosts showing the characteristic brûlé. These truffle-grounds were among those surveyed during previous investigations in 2006 [7], [8].

## Materials and Methods

### Soil Sampling

Four productive truffle-grounds of *Tuber melanosporum*/*Quercus pubescens* were selected for sampling. They showed the characteristic brûlé of several meters and belonged to La Station de la trufficulture de Cahors-Le Montat, France (Table S1). No specific permits were required for the described field studies. These grounds are managed by Lycée professionnel agricole Lacoste, Le Montat, which is directed by Pierre Sourzat in collaboration with truffle-ground owners. The field studies did not involve endangered or protected species. All four truffle-grounds were among the sites surveyed during the previous investigations [7], [8]. For each truffle-ground, the same brûlé investigated previously was sampled. Samples were collected in March 2008. Each of the four brûlés sampled had a different shape and size, as described and illustrated previously [7]; 12 samples were collected from inside and 12 from outside of a brûlé, resulting in a total of 96 samples. Soil samples were taken approximately to a depth of 10–15 cm and stored at 4°C. Soil chemical parameters for the truffle-grounds investigated in the present work by DGGE and microarray analyses are presented in Table S1.

### Soil DNA Extraction

The 12 samples coming from the same brûlé were grouped into four pools (three samples/pool). In this way, four replicate pools were obtained for each brûlé and four from outside each brûlé, resulting in eight samples per site. Since there were four brûlé sampled, 16 pools from the brûlé areas and 16 from the corresponding outside areas were obtained, resulting in 32 soil pools. All 32 pools were air-dried and sieved through a 2-mm sieve to homogenize the soil samples as much as possible. Total DNA was extracted for each pool from 0.5 g of soil using Fast DNA Spin Kit for Soil (MPBiomedicals), and cleaned with UltraClean kit (Mobio). The DNA concentration of each pool was measured on a 1% agarose gel. The same DNA amount was employed in the following analyses. All the samples were analyzed by DGGE and one truffle-ground also by phylogenetic microarrays.

### Denaturing Gradient Gel Electrophoresis Analysis

The 32 DNA pools coming from the above-mentioned four truffle-grounds were subjected to nested or seminested PCR to amplify 16S rRNA genes of *Alphaproteobacteria*, *Betaproteobacteria*, *Actinobacteria*, *Pseudomonas* or *Bacillus* as previously described [16], [17], [18], [19], [20], [21] (See Table 1 for primers and PCR conditions). PCR products were analyzed by DGGE. DGGE of the 16S rRNA gene amplicons was performed according to Gomes et al. [22] and the gel was silver stained [23]. GelCompar II 6.5 was used to analyze different microbial DGGE profiles. To test whether there were significant differences between bacterial communities located inside and outside the brûlés, the pairwise Pearson correlation index was subjected to PERMTEST [24].

**Table 1:** Primers and PCR conditions employed in all the first-round PCR performed for DGGE analysis in this study. The first-round amplifications are specific for each of the bacterial groups considered: *Actinobacteria*, *Alphaproteobacteria*, *Betaproteobacteria*, *Pseudomonas* and *Bacillus*.

Target	*Alphaproteobacteria*	*Betaproteobacteria*	*Actinobacteria*	*Pseudomonas*	*Bacillus*
Primers	R1492 (Weisburg et al., 1991)/F203α (Gomes et al., 2001)	R1492/F948β (Gomes et al., 2001)	L1401 (Nubel et al., 1996)/F243HGC (Heuer et al., 1997)	F311Ps/R1453Ps (Milling et al., 2004)	BacF (Garbeva et al., 2003)/R1378 (Heuer et al., 1997)
Initial denaturation	94°C 7 min	94°C 10 min	94°C 5 min	94°C 7 min	94°C 5 min
Denaturation	94°C 1 min	94°C 0.5 min	94°C 1 min	94°C 1 min	94°C 1 min
Annealing	56°C 1 min	64°C 2 min	63°C 1 min	63°C 2 min	65°C 1 min 30″
Elongation	72°C 2 min	72°C 1 min	72°C 2 min	72°C 2 min	72°C 2 min
Final elongation	72°C 10 min	72°C 10 min	72°C 10 min	72°C 10 min	72°C 10 min

### PhyloChip Analysis

DNA microarray analysis was applied to a single truffle-ground, Brûlé 1. Three DNA pools from this brûlé and three from outside were used as replicate samples. From each pool, 16S rRNA genes were amplified using an 8-temperature gradient PCR and universal 16S rRNA gene primers for bacterial and archaeal amplification respectively (27f 5′- AGAGTTTGATCCTGGCTCAG -3′, 4fa 5′- TCCGGTTGATCCTGCCRG -3′, and the reverse 1492r 5′- GGTTACCTTGTTACGACTT -3′ for both forward primers). All DNA templates were diluted 1∶5 before setting up PCR for bacterial amplification and were diluted 1∶10 before setting up PCR for archaeal products. One microliter of diluted template was used in each tube in the PCR gradient. 25 µL reactions contained final concentrations as follows: 1×TITANIUM™ Taq Buffer with 2 mM MgCl_2_, 300 nM each primer, 200 µM each dNTP (Promega) (and where thymidine and uracil were used in a ratio of 2∶1 to yield a combined total of 200 µM), 1 µg/uL bovine serum albumin (Roche Applied Science, Indianapolis, IN), and 1×Titanium Taq DNA polymerase (Clontech Laboratories, Inc, Mountain View, CA). Reactions were amplified using an iCycler (Bio-Rad, Hercules, CA) and the following thermocycling conditions: 95°C for 3 min for initial denaturation, 25 cycles (bacteria) of 95°C for 30 sec, 48–58°C for 30 sec, and 68°C for 2 min, and then final extension for 10 min at 68°C. The archaea were amplified for 30 cycles instead of 25 cycles. PCR products from each annealing temperature for a sample were combined and concentrated to 40 µL or less final volume using Microcon YM-100 filters (Millipore, Billerica, MA). Forty microliters water were added to the filter units and spun through prior to loading the PCR product for concentration. 1 µL of concentrated PCR product was quantified on a 2% agarose E-gel using the Low Range Quantitative DNA Ladder (Invitrogen, Carlsbad, CA). Because the archaeal primers also amplify eukaryotic DNA, the archaeal PCR products were gel-purified to exclude unspecific eukaryotic amplicons and recovered using a MinElute Gel Extraction kit (Qiagen, Valencia, CA).

Five hundred ng of bacterial PCR product and 100 ng of archaeal PCR product were prepared for G3 PhyloChip microarray analysis using the GeneChip WT Double Stranded DNA Terminal Labeling Kit, Control Oligo B2, and the GeneChip Hybridization, Wash, and Stain Kit (Affymetrix, Santa Clara, CA) per the manufacturer’s instructions with the following optimizations for the custom microarray. The fragmentation reaction consisted of the PCR product, 3.0 µL of 10×Fragmentation Buffer, 0.94 µL UDG (10 U/µL), 1.41 µL APE (100 U/µL), 202 ng of an internal standard (mixture of known amplicons), and water to a final volume of 30 µL. The reaction was incubated in a thermal cycler at 37°C for one hour, 93°C for 2 min, and then 4°C for 10 min. The fragmented product was biotinylated using 8 µL 5×TdT buffer, 1.34 µL TdT enzyme, and 0.66 µL GeneChip Labeling Reagent in a final volume of 40 µL, incubated at 37°C for one hour, 70°C for 10 min, and then 4°C for 10 min. Two microliters of 0.5 M EDTA was added to stop all enzymatic reactions. The product was prepared for hybridization onto the array by adding 2.2 µL Control Oligo B2, 65 µL 2×Hybe Mix, 20.4 µL DMSO, and sufficient water to bring the final volume to 130 µL. This mixture was incubated at 99°C for 5 min to denature the DNA strands and then held at 48°C for 5 min before being transferred to a pre-hybridized array also being held at 48°C. Arrays were hybridized at 48°C and 60 RPM overnight and then washed and stained using procedures recommended by Affymetrix. Details of washing, staining, image-capture, and initial data processing have been described previously [25]. Fluorescence intensities of the internal standards were used to normalize total array intensities among samples. The PhyloChip has been annotated according to the Greengenes taxonomy [26].

### PhyloChip Data Analysis

The OTUs were created as groups of 16S rRNA genes from the public database sharing 99.5% average similarity, and were arranged into 92 phyla, 730 families, and 1,996 genera [27]. Bacterial OTUs were analyzed similarly to that described in Hazen et al. [25]. Stage1 cutoffs used to establish presence/absence calls were set as follows using an updated taxonomy [26], [27], as applied to PhyloChip OTUs: pairs_counted > = 7, pairs_scored> = 7, q1> = 0.5, q2> = 0.93, and q3> = 0.98. OTUs passing Stage1 cutoffs were examined for cross-hybe potential (i.e., non-specific hybridization) among probes in neighboring probe sets (OTUs). After a penalty was assessed for potential cross-hybe, the new q3 values were used to establish presence/absence for species: q3> = 0.1. All OTUs in the passing species were then used for further analysis. The probe intensities of the ‘spiked’ amplicons were used to scale intensities for the rest of the probes in a sample to allow comparisons across samples.

To track populations of archaeal OTUs that were potentially dissimilar from those present in the public databases, the criteria were loosened. An archael OTU was considered present when Stage1 q2 values were > = 0.8 and q3 values were > = 0.9. Only those OTUs with at least one sample having a trimmed mean intensity (mean with the highest and lowest values removed before averaging) of at least 1000 units were considered for q2 and q3 cutoffs.

PhyloChip data were used to compare the richness (the number of passing OTUs) of samples collected inside to that of samples collected outside the brûlé (referred to hereafter as relative richness) and abundance of OTUs (considered to be reflected by the signal intensity of an OTU) when compared across samples (not within a sample and not considered to be an absolute value, and so hereafter referred to as relative abundance). Community structure is based on the number and intensity of OTUs. Significant effects of niche on the relative diversity in terms of the number of detected OTUs for different bacterial phyla were tested by multiple-t tests. Discriminative taxa (taxonomic groups with a large proportion of OTUs with significantly different signal intensities) between inside and outside the brûlé were identified, as recently described [28]. Briefly, the fluorescent signal intensity of each OTU was log10 transformed, centered (to reach a 0 mean), standardized (to reach a SD of 1) and assigned to different taxonomic groups. To identify discriminative OTUs between inside and outside the brûlé, multiple-t tests of log transformed normalized signal intensities for each OTU were applied using the software package R (2.11.0). Differences in community structure at different taxonomic levels such as phylum, class, order, and family between inside and outside the brûlé were tested using a newly developed method based on the first three principle components [29]. These adjusted data were the basis for PCA using the ‘FactoMineR’ function of the R package. OTUs differing significantly (unadjusted *p*<0.05) and having 1.8-fold differences in mean intensity between inside and outside the brûlé were used to make heatmaps. Heatmaps were generated using untransformed intensity data and the R package ‘gplots’ [30]. CEL files of the scanned microarray images and scaled OTU intensity data for each sample are available at http://greengenes.lbl.gov/Download/Microarray_Data. The files specific for this study are in a zipped file called Mello_2013_PLoS_ONE.zip.

## Results

### Denaturing Gradient Gel Electrophoresis Analysis

DGGE was applied to all the sample pools in order to have a comprehensive picture of the soil bacterial composition inside and outside the four brûlés investigated. DGGE of *Actinobacteria*, *Alphaproteobacteria, Betaproteobacteria* and *Bacillus* were characterized by a large number of bands in each lane. For these bacterial groups, the profiles of the four replicates were highly reproducible with very similar banding patterns, demonstrating the representativeness of each composite sample. DGGE of *Pseudomonas* showed a smaller number of bands and a greater variability among the replicates. To compare the soil bacterial community compositions between inside and outside the brûlés, DGGE profiles for *Actinobacteria*, *Alphaproteobacteria*, and *Pseudomonas* were clustered by UPGMA based on Pearson correlation indices. DGGE profiles for *Betaproteobacteria* and *Bacillus* were excluded from this analysis because one lane in each gel was missing. The profiles of the four replicates from the same brûlé (or those from the paired outside soil) were highly similar (>83% similarity on average) for *Actinobacteria* and *Alphaproteobacteria*, whereas they showed only 34–72% similarity for *Pseudomonas*, with the exception of outside samples at Brûlé 3, which had 85% similarity. Permutation analyses revealed that there were significant differences in community compositions among the brûlés studied (Table 2). Thus, comparisons were performed between inside vs. outside samples for each brûlé. In general, the community compositions of all three bacterial groups studied were significantly different inside vs. outside the brûlés (Table 2). Therefore, the community composition of *Actinobacteria* and *Pseudomonas* was affected at all four studied brûlés, but that of *Alphaproteobacteria* only slightly at Brûlé 4 (Table 2).

**Table 2:** Percent dissimilarity of microbial DGGE fingerprints for different taxa inside *versus* outside the brûlés or among the four brûlés.

		*Actinobacteria*	*Alphaproteobacteria*	*Pseudomonas*
Inside vsOutside	Total	12.6*	1.9*	12.9*
	Brûlé1	10.8*	0.9	17.1*
	Brûlé2	13.7*	0.8	11.5*
	Brûlé3	14.2*	2	7.3*
	Brûlé4	11.5*	3.7*	15.5*
Among brûlés		21.4*	12.4*	21.3*

Note: * : significant (p<0.05) difference between treatments as revealed by 1000 times permutation tests.

### PhyloChip Analysis

Phylochip analysis was performed for an in-depth investigation of bacterial and archaeal communities inside and outside one brûlé of a *T. melanosporum/Quercus pubescens* truffle-ground, (Brûlé 1, Table S1). Altogether, 12,837 OTUs, grouped into 1370 genus-level clusters, were called present in at least one of six samples (Table 3). There was a high degree of overlap of OTUs called present inside and outside the brûlé: more than 91. % of the OTUs were detected both inside and outside the brûlé, while 32.4% of the OTUs were detected in all six samples. The majority belonged to Domain Bacteria (>99.9%), while only 14 OTUs were found for Archaea (Table 3). Although gene amplification using archaeal primers yielded a large amount of product, only a few archaeal OTUs passed the criteria to be considered present, so our understanding of the interior and exterior areas of the brûlé may be limited still. There may be taxa present that were not amplified by the primers we employed.

**Table 3:** Number of OTUs detected for soil samples collected inside the brûlé vs. outside the brûlé using PhyloChip analysis.

Domain	Phylum	*INSIDE*	*OUTSIDE*	*Total*
Bacteria	Proteobacteria	*3298±226*	*3123±702*	*5187*
	Actinobacteria	*2534±114*	*2219±313*	*3237*
	Firmicutes	***1025±117***	*629±202*	*1764*
	Bacteroidetes	*393±11*	*595±141*	*920*
	Acidobacteria	*312±14*	*276±49*	*392*
	Planctomycetes	*161±9*	*148±29*	*205*
	Verrucomicrobia	*142±22*	*129±20*	*200*
	Chloroflexi	*133±8*	*114±16*	*182*
	Cyanobacteria	***89±7***	*48±17*	*132*
	Gemmatimonadetes	*84±3*	*79±11*	*99*
	Spirochaetes	*37±5*	*32±12*	*60*
	Tenericutes	*28±4*	*26±12*	*60*
	others	*275±20*	*236±44*	*385*
Archaea		*10±3*	*8±1*	*14*
Sum		*8522±392*	*7661±1528*	*12837*

Note: Bold number: significantly higher (p<0.05) number of OTUs detected inside the brûlé compared to outside. INSIDE and OUTSIDE: average number of OTU ± standard deviation.

The bacterial OTUs were affiliated to 75 phyla. Among them, *Proteobacteria* (5,187 OTUs), *Actinobacteria* (3,237 OTUs), *Firmicutes* (1,764 OTUs), *Bacteroidetes* (920 OTUs), *Acidobacteria* (392 OTUs), *Planctomycetes* (205 OTUs), *Verrucomicrobia* (200 OTUs), *Chloroflexi* (182 OTUs) and *Cyanobacteria* (132 OTUs) were detected (Table 3). Although the total number of detected OTUs was not significantly different inside vs. outside the brûlé, more variation among biological replicates (greater standard deviation) was found for replicate pools from outside (Table 3). At the phylum level, the relative richness of *Firmicutes* and *Cyanobacteria* (in terms of number of OTU) was greater for samples from inside than from outside (Table 3). Although not statistically significant, the trend of *Bacteroidetes* was opposite (Table 3). This trend may be explained by comparing the relative richness at the level of class, where the richness of Class *Flavobacteria* (within Phylum *Bacteroidetes*) was greater for samples from outside than from inside (p<0.05).

### Taxa with a High Proportion of Discriminative OTUs

Multiple t-tests were applied to identify discriminative OTUs based on intensity differences for the brûlé interior vs. exterior (unadjusted *p*<0.05), and taxa with a high proportion of discriminative OTUs are summarized in Table 4. More than 15% of bacterial OTUs detected were significantly different inside vs. outside the brûlé; by contrast, only one of 14 OTUs belonging to archaea had significantly different signal intensities inside vs. outside the brûlé. On the basis of such results, we suggest that the archaea detected may play a secondary role in the truffle ecosystem, though more inclusive primers should be applied before reaching final conclusions about the role of archaea.

**Table 4:** Discriminative taxa between inside (IN) and outside (OUT) the brûlé determined by PhyloChip analysis.

Phylum	Class	Order	Family	Genus	IN	OUT	Total
*Proteobacteria*	*Betaproteobacteria*	*Burkholderiales*	*Oxalobacteraceae*	***Massilia***	12	0	29
	*Gammaproteobacteria*	***Pseudomonadales***	***Pseudomonadaceae***	***Pseudomonas***	0	235	619
	*Deltaproteobacteria*	*Myxococcales*	***Cystobacteraceae***		7	0	20
			***Myxococcaceae***	***Myxococcus***	9	0	33
*Actinobacteria*	*Actinobacteria*	*Actinomycetales*	*Actinomycetaceae*	***Actinomyces***	4	0	20
			*Actinosynnemataceae*	***Saccharothrix***	4	0	13
			***Frankiaceae***	***Frankia***	3	0	15
			***Geodermatophilaceae***	***Geodermatophilus***	7	0	14
			***Microbacteriaceae***	***Microbacterium***	0	33	142
			***Micrococcaceae***	***Arthrobacter***	32	5	161
			***Micromonosporaceae***	***Micromonospora***	37	0	96
			***Mycobacteriaceae***	***Mycobacterium***	25	2	183
			***Pseudonocardiaceae***	***Pseudonocardia***	8	0	30
			***Streptosporangiaceae***	***Streptosporangium***	5	0	14
		***MC47***			16	0	73
		***Rubrobacterales***	***Rubrobacteraceae***	***Rubrobacter***	7	0	14
		*Solirubrobacterales*	***Conexibacteraceae***		5	0	30
***Firmicutes***	***Bacilli***	***Bacillales***	***Bacillaceae***	***Bacillus***	375	1	554
				***Geobacillus***	4	0	24
*Acidobacteria*	***Chloracidobacteria***				11	0	43
***Bacteroidetes***	***Flavobacteria***	***Flavobacteriales***	***Flavobacteriaceae***	***Chryseobacterium***	0	48	59
				***Flavobacterium***	0	56	114
				***Riemerella***	0	11	13
	*Sphingobacteria*	***Sphingobacteriales***	***Sphingobacteriaceae***	***Pedobacter***	0	12	15
***Cyanobacteria***	***Chloroplast***				9	1	49
	***Oscillatoriophycideae***	***Oscillatoriales***	***Phormidiaceae***		9	0	21
	***Synechococcophycideae***	***Synechococcales***	***Synechococcaceae***	***Prochlorococcus***	4	0	13
*Gemmatimonadetes*	*Gemmatimonadetes*	***Gemmatimonadales***	***Gemmatimonadaceae***		23	0	42
*Nitrospirae*	*Nitrospira*	*Nitrospirales*	***Nitrospiraceae***	***Nitrospira***	5	0	20

Bold text: Taxa with significantly different community structures between inside (IN) and outside (OUT) the brûlé. IN and OUT: number of OTUs with significantly different signal intensities inside vs. outside. Total: total number of OTUs detected for the taxa.

Among the discriminative bacterial OTUs, 8.7% had significantly higher signal intensities within the brûlé compared to outside of it. The majority (>88%) of these OTUs were affiliated to the phyla *Firmicutes* (484 OTUs), *Actinobacteria* (341 OTUs) and *Proteobacteria* (160 OTUs). Large proportions of OTUs belonging to the family *Gemmatimonadaceae* (55%) or genera *Bacillus* (68%), *Geodermatophilus* (50%), *Rubrobacter* (50%), and *Massilia* (41%) had significantly higher signal intensities inside compared to outside the brûlé, indicating that the brûlé was enriched with these groups, while several but fewer OTUs (6.7%) had significantly lower signal intensities inside than outside the brûlé. Most of the OTU with lower intensities were found in the phyla *Bacteroidetes* (378 OTUs), *Proteobacteria* (324 OTUs) and *Actinobacteria* (80 OTUs). A large proportion of OTUs belonging to the genera *Riemerella* (85%), *Chryseobacterium* (81%), *Pedobacter* (80%), *Flavobacterium* (49%) and *Pseudomonas* (40%) had significantly lower signal intensities inside compared to outside the brûlé, suggesting that these taxa are less abundant inside the brûlé.

For selected genera, heatmaps were generated to view species-level responses. The following species within *Bacillus* had greater relative abundance inside than outside the brûlé: *B. foraminis,B. acidicola, B. clausii, B. amyloliquefaciens, B. flexus, B. malacitensis, B. muralis, B. cytotoxicus, B. anthracis, B. soli, B. asahii, B. badius, B. sonorensis, B. gelatini, B. safensis, B. firmus, B. longiquaesitum, B. weihenstephanensis, B. funiculus, B. humi, B. nealsonii, B. agaradhaerens, B. barbaricus, B. pseudofirmus, B. algicola, B. fumarioli, B. halmapalus, B. hwajinpoensis* (Figure S1). Conversely, *R. anatipestifer* and *R*. *columbina* within *Riemerella* (Figure S2), *C. indologenes, C. gleum, C. luteum* and*C. daecheongense* within *Chryseobacterium* (Figure S3), *P. cryoconitis* within *Pedobacter* (Figure S4), *F. succinicans, F. xanthum, F. psychrophilum, F. frigidarium, F. dentifricans, F. columnare,* within *Flavobacterium* (Figure S5), and *P. veronii, P. oryzihabitans, syringae group genomosp, P. savastanoi, P. nitroreducens, P. chororaphis, P. umsongensis, P. entomophila, P. stutzeri, P. anguilliseptica, P. koreensis, P. libanensis, P. mandelii, P. borbori, P. rhodesiae, P. syringae pv. coryli, P. cedrina, P. mediterranea, P. mosselii, P. rhizospherae, P. mendocina* within *Pseudomonas* had greater relative abundance outside (Figure S6).

### Taxa with Discriminative Community Structure

**Figure 1:**
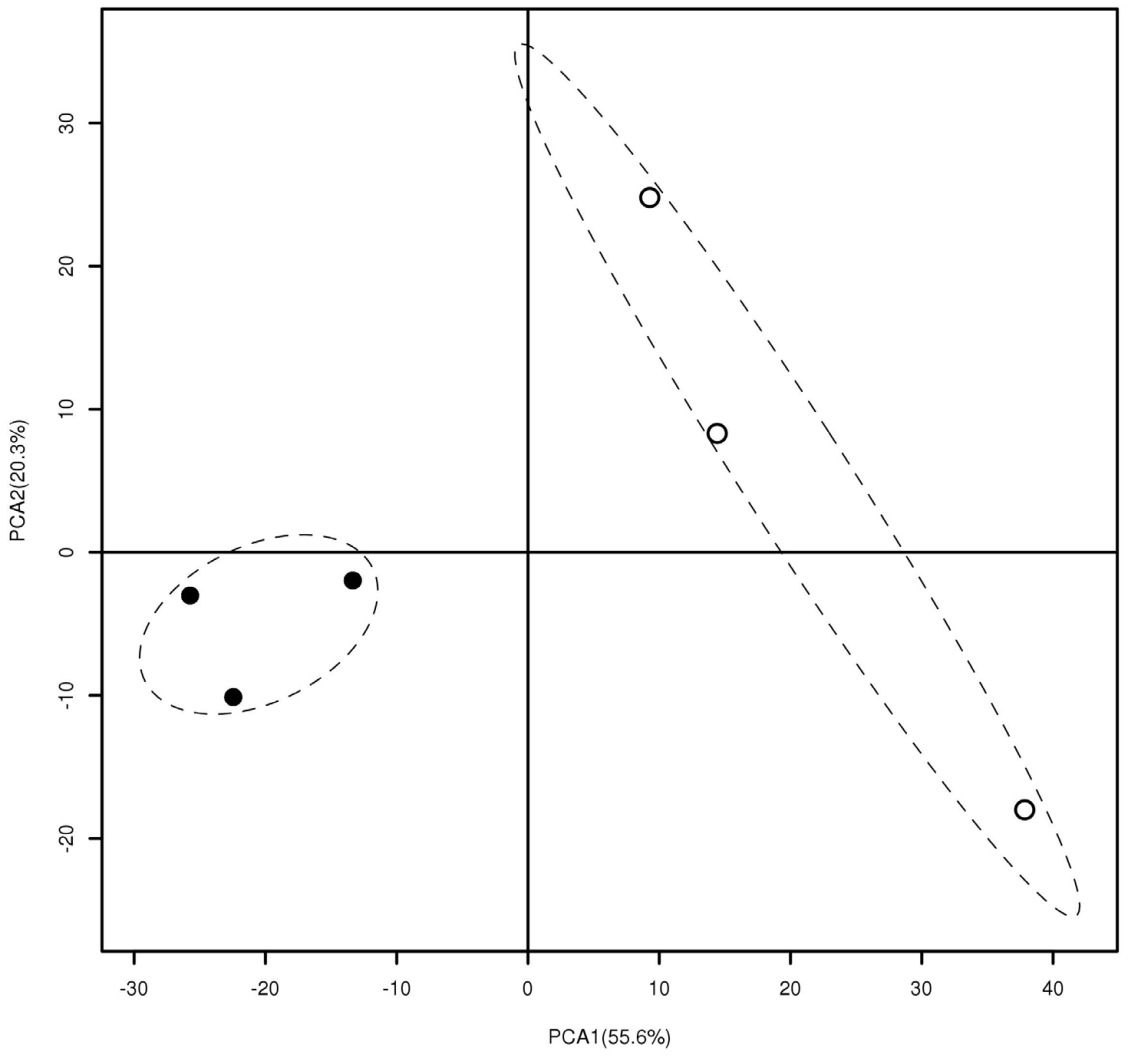
Principal component analysis of PhyloChip data from ***Bacteroidetes*** **inside (solid dots) or outside (empty dots) the brûlé.** The first and second principal components explain 56% and 20% of total variance.

To compare the community structure between inside and outside the brûlé, different taxonomic groups (from domain to family) were analyzed using a principal component-based test. This procedure provides a sensitive analysis of the differences of those OTUs with low signal intensities. The result suggests that *Firmicutes, Bacteroidetes* and *Cyanobacteria* differed significantly in community structure between the two niches (Table 4, Figure 1). Significant difference in community structure between inside and outside the brûlé was also found for several genera such as *Massilia, Pseudomonas, Myxococcus, Actinomyces, Saccharothrix, Frankia, Geodermatophilus, Microbacterium, Arthrobacter, Micromonospora, Pseudonocardia, Streptosporangium, Rubrobacter, Bacillus, Geobacillus, Chryseobacterium, Flavobacterium, Riemerella, Pedobacter, Prochlorococcus, Nitrospira* (Table 4).

## Discussion

The production of a brûlé is an impressive effect of *T. melanosporum* on the surrounding environment: its causes and mechanisms are only partially understood because few studies have focused on them. Here two molecular techniques - DGGE and PhyloChip - were used to compare bacterial community structures between *T. melanosporum* brûlés and surrounding soils. Both analyses detected differences in community composition inside compared with outside a brûlé. DGGE analysis, which was applied to four brûlés, revealed differences in composition for a few taxa (*Actinobacteria*, *Alphaproteobacteria,* and *Pseudomonas,* this last within the *Gammaproteobacteria*). PhyloChip analysis, which was applied to only one brûlé, revealed striking differences in OTU abundance and for a larger number of taxa. In the current study, *Pseudomonads* and most members of *Actinobacteria* had different abundances according to both DGGE and PhyloChip microarray analyses. This concordance of results on a complex matrix such as soil lends strength to the findings. The results from PhyloChip microarray analysis of a lower abundance of *Pseudomonads* and of higher abundance of all the genera of *Actinobacteria,* except *Microbacterium*, inside compared to outside the brûlé, suggest a close relationship between the *Actinobacteria* and brûlé formation.

### DGGE Profiles Reflect Differences in Microbial Community Composition Inside vs. Outside the Brûlés

DGGE analysis revealed that the profiles of the four replicates were highly similar within each brûlé but distinct between samples collected inside and outside of the brûlés, indicating that certain bacterial populations were selected within the brûlé. The more minor distinctions found among the brûlés may be related to the indigenous soil bacterial communities from the different truffle-grounds, resulting, for instance, in a significant difference in the community composition of *Alphaproteobacteria* being observed only for Brûlé 4, though the distinctions also simply may be due to individual variability of brûlés, (i.e., variability among brûlés within the same truffle-ground). In contrast to *Alphaproteobacteria*, the community compositions of *Actinobacteria* and *Pseudomonas* were significantly different inside vs. outside the brûlé at all four studied brûlés, suggesting that the selected bacteria belonging to *Actinobacteria* or *Pseudomonas* respond to the conditions specific to the brûlé, which supports it as a well-structured ecological niche.

The complex band patterns observed in *Actinobacteria, Alphaproteobacteria*, *Betaproteobacteria* and *Bacillus* profiles mirrors the high diversity for these bacterial groups. This great biodiversity, as expected in a natural soil [31], results in too many populations for each to be reflected in a band, and so the bands observed represent only the dominant types and not the total richness of a soil sample [32]. This complexity prevented us from seeing many differences in relative richness inside vs. outside the brûlés using DGGE. By contrast, the lower number of bands observed for *Pseudomonas* spp. profiles reflect a lower richness in soil (or perhaps a greater dominance of a few *Pseudomonads*). The greater variability of fingerprints observed across sample pools for *Pseudomonas* spp. may depend on the low quantity of target DNA of the first specific PCR of *Pseudomonas* spp. [33]. This also may indicate that DGGE, in conjunction with the specific enrichment of targeted groups via nested PCR, is sensitive enough to detect differences in this group (reported to constitute approximately 1% of the bacterial communities in soil) with low relative abundance.

### PhyloChip Analysis Opens a Window on the Microbial Biodiversity within the Brûlé

The apparent diversity was very high, as expected for soil samples. A total of 12,837 OTUs were detected according to the Greengenes taxonomy, which has been shown to provide great taxonomic resolution of the 16S rRNA gene reference sets [26]. OTUs belonging to the *Proteobacteria* were the most frequently detected OTUs, which is similar to findings for plant-associated soils [34] and is not unexpected, as there are more probes on the G3 PhyloChip for *Proteobacteria* than for other phyla. The finer resolution of the microarray with respect to DGGE analysis allowed more subtle differences to be noted. Only small differences in richness at the phylum level have been found (*Firmicutes* and *Cyanobacteria* showed higher relative richness inside the brûlé). Given the breadth and depth of microbial communities in soils, it is perhaps not surprising that there were so few differences observed between the two areas. Since *Cyanobacteria* utilize the energy of sunlight to drive photosynthesis, it is possible they take advantage of access to more light provided by inhibition of the understory in the brûlé. The absence of herbaceous vegetation inside the brûlé also may explain why the relative abundances of *Firmicutes* - which are, among all bacteria, the least dependent on rhizodeposition [35] - were higher inside than outside the brûlé.

A large proportion of OTUs belonging to *Bacillus* (68%), *Gemmatimonadaceae* (55%), *Geodermatophilus* (50%), *Rubrobacter* (50%), and *Massilia* (41%) had significantly higher signal intensities inside vs. outside the brûlé, while relatively large proportions of OTUs belonging to *Riemerella* (85%), *Chryseobacterium* (81%), *Pedobacter* (80%), *Flavobacterium* (49%) and *Pseudomonas* (40%) had significantly higher signal intensities outside compared to inside the brûlé. *T. melanosporum* fruiting bodies are generally collected inside the brûlé. While the relationship between *T. melanosporum* and other fungi present in the brûlé has been shown recently [7], [8], no data were available on the relationship between this fungus and the bacteria inhabiting the brûlé. The findings acquired in the present study do suggest the presence of a relationship.

*T. melanosporum* acts as the dominant fungus in the brûlé investigated in the present study, inhibiting the other ectomycorrhizal fungi, and consequently changing the fungal community structure inside the brûlé [7], [8]. Moreover, annual brûlé expansion causes deep changes in the roots of the host plant, in the plant hormonal balance, and a marked aggressive competition zone located at the brûlé periphery [36], [37], [9]. In addition, there are expected to be biologically relevant changes in light exposure and in moisture levels. This niche formation is affected by *T. melanosporum* through the emission of diffusible toxic metabolites, recently recognized as allelochemicals. Phenolic compounds represent an important group of allelochemicals distributed in soils and are reported to be more highly concentrated in brûlés [38]; the measurement of their higher concentrations in the most aggressive area of the brûlé is a further demonstration of the close relationship between *T. melanosporum* allelopathy and brûlé formation [9].

The different signal intensities we have found for members of bacteria between inside and outside the brûlé are the first demonstration, to our knowledge, that not only fungal communities but also other microorganisms are effected in the brûlé. It is now accepted that bacteria are involved in tripartite interactions with plants and mycorrhizal fungi in which the release of active molecules, including volatiles, and physical contact among the partners seem important [39]. Some bacteria called mycorrhiza helper bacteria (MHB) promote the formation of symbioses and complement the functions of mycorrhizas, such as nutrient uptake and biological control of host plants [40]. Because truffles arise from soil, they trap bacteria, yeasts and filamentous fungi [41], [42], [43]. It is known from data on the bacterial communities present in truffle fruiting bodies there are members belonging to *Proteobacteria*, *Firmicutes* (*Bacillaceae)* and *Bacteroidetes, and* the predominant components obtained through molecular investigations are *Alphaproteobacteria* (mainly members of the *Sinorhizobium*, *Rhizobium* and *Bradyrhizobium*), whereas the cultivable fraction is represented by *Gammaproteobacteria*, which are mostly fluorescent pseudomonads [10]. The study just mentioned, however, was not conducted on *T. melanosporum* fruiting bodies but instead on the bacterial communities present in the white truffle, *T. magnatum*. Along with the phylogenetic analysis, nitrogenase gene expression and activity have been demonstrated, suggesting that *T. magnatum* fruiting bodies may host bacterial-driven nitrogen fixation events [44]. In addition to this, growth-stimulating strains of fluorescent pseudomonads have been identified in fruiting bodies of *T. borchii* and of other ectomycorrhizal fungi, and a bacterium belonging to *Cytophaga-Flexibacter-Bacteroides* group has been associated with *T. borchii* mycelium [45], [46]. Fluorescent pseudomonads are regarded as MHB, and therefore can promote the establishment of the symbiosis between the mycelium and its hosts before becoming part of the fruiting body bacterial community [12], though we saw no evidence that such a relationship might be active in the *T. melanosporum* brûlé, as the OTU in the genus *Pseudomonas* had higher relative abundances outside the brûlé than inside.

While important, the previous works on truffle microbial communities employed truffle species other than *T. melanosporum*, so the microbial community resident in *T. melanosporum* fruiting bodies remains largely unknown. Among all the species in the present study showing significant differences between inside and outside of the brûlé, none was found in the bacterial communities of the previously investigated truffle species, the white truffles *T. magnatum* and *T. borchii*.

It is known that white truffles host yeasts and filamentous fungi, in addition to bacteria, among their gleba hyphae or at the surface of the peridium [43], supporting a large microbiome. Even though at the moment we have no data on the black truffle microbiome, we suggest that such a potential *T. melanosporum* microbiome mirrors, in some way, the microbes inhabiting the brûlé, creating a continuity between the soil brûlé communities and those thriving inside the fruiting bodies. As an alternative, truffles may select some microbes, offering a specific niche for some populations. Both scenarios will likely result in different abundance between inside and outside the brûlé, as we report. In addition, we hypothesize that different species of truffles may sequester selected bacterial taxa, changing their abundance in the brûlé. Since each truffle species is associated with given soil physical-chemical parameters, these parameters also could affect the composition of the bacterial community in soil and in truffles. The future analysis of the *T. melanosporum* microbiome could open the possibility to link the community present in the fruiting body to that resident in the brûlé.

### Some Bacterial Groups may Provide Specific Ecological Services within the Brûlé

Dimethylsulfide (DMS) is a principal volatile compound released by *T. melanosporum* [47] and so we wondered whether among the bacteria in higher relative abundance in the brûlé there were any known DMS-degrading bacteria. Current knowledge on the microbial cycling of DMS reports that the microorganisms able to degrade DMS have been isolated from a wide array of environments, from seawater to thermophilic fermenter sludge, and vent gas of the pulp and paper industry, though rarely from soil [48], [49]. Despite this, we found members of two genera, *Arthrobacter* and *Pseudonocardia,* listed by Schäfer et al. as having members that could degrade DMS [49], that were more prevalent within the brûlé than outside. *Arthrobacter sulfonivorans* and *A*. *methylotrophus* have been isolated from an environment similar to ours, namely rhizospheres associated with *Allium* and *Tagetes,* whereas *Pseudonocardia sulfidoxydans* and *P*. *asaccharolytica* were isolated from a DMS-producing animal-rendering plant biofilter. We think that with further investigation of the soil for the isolation of DMS-degrading bacteria, there could be further correlations between the bacteria in the brûlé and their potential ability to degrade DMS.

Additionally, it is known that volatile sulfur compounds control soil borne phytopathogenic fungi in crop plant ecosystems. Microbial consortia recently have been identified as markers of these disease-suppressive soils [34]. Since the brûlé is very poor in vegetation, root exudates are expected to be less abundant than they would be outside the brûlé, being limited to the roots of the host plant. It is known that microbes are attracted by root exudates in the rhizosphere, and recently it has been demonstrated how the microbiome of *Arabidopsis thaliana* changes from the external rhizosphere to the endophytic compartment [50]. The results from our survey demonstrate some quantitative differences in the OTU numbers. This observation suggests that root exudates from grass plants likely are not the main drivers of bacterial attraction in the truffle niche, while they could be another biotic factor that explains the qualitative differences between outside (abundant root exudates) and inside (limited root exudates) the brûlé. Following these hypotheses, we hope in the future to detect the connections that link bacterial diversity to the multiple factors controlling the brûlé ecosystem.

### Relationship between Diversity Changes and Detection Limits

The purpose of the present study was to compare the bacterial and archaeal communities from areas inside and outside of the brûlé of *T. melanosporum.* Several taxonomic groups that had high proportions of discriminative OTUs were identified. Although our investigation was conducted in a rigorous and consistent way, primer bias is inescapable. Some taxa may not have been detected. And too, in the case of microarrays technology, we are limited to estimating relative richness and abundance differences among groups of samples, though other methods also have limitations. Our investigation relied on directly-extracted DNA, which includes DNA that can remain in the environment after cell death [51]. RNA-based studies would have provided information about the more active microbes in the samples at the time of collection, though such studies may have missed less active members that might be active at other times of year. Nevertheless, since our study is based on the comparison of microbial communities from two areas, we think such biases likely would have been similar for samples collected inside and outside the brûlés. Thanks to the recent advances in the analysis of functional genes in environmental samples, large-scale (next generation) sequencing, and RNA-centered meta-transcriptomic methods, both structure and function of the active microbial community of the brûlé could be elucidated further in the future.

### Conclusions

This is the first report to compare the bacterial and archaeal 16S rRNA genes from areas inside and outside of the brûlé of *T. melanosporum growing in* association with *Quercus pubescens*. PhyloChip and DGGE analyses revealed differences between inside and outside the brûlé for some taxa. The different signal intensities measured for members of bacteria and archaea inside vs. outside the brûlé are the first demonstration, to our knowledge, that not only fungal communities but also other microorganisms are affected by the brûlé. Findings of significantly increased OTUs inside the brûlé should be confirmed for more sites and perhaps by other methods (e.g. by qPCR and HiSeq sequencing), and may contribute to future searches for microbial bio-indicators of the brûlé. Understanding the ecological role of these brûlé associated bacteria and how they interact with *T. melanosporum* is the next step.

## Supporting Information

Figure S1 **Heatmap of the OTUs that were both significantly different and had nearly a 2-fold difference in average intensity between inside and outside the brûlé for** ***Bacillus*** **.** In_1, In_2, In_3 and Out_1, Out_2, Out_3, respectively, were pools from inside and outside the brûlé and were used as replicate samples.
(EPS)

Figure S2 **Heatmap of the OTUs that were both significantly different and had nearly a 2-fold difference in average intensity between inside and outside the brûlé for** ***Riemerella*** **.** In_1, In_2, In_3 and Out_1, Out_2, Out_3, respectively, were pools from inside and outside the brûlé and were used as replicate samples.
(PDF)

Figure S3 **Heatmap of the OTUs that were both significantly different and had nearly a 2-fold difference in average intensity between inside and outside the brûlé for** ***Chryseobacterium*** **.** In_1, In_2, In_3 and Out_1, Out_2, Out_3, respectively, were pools from inside and outside the brûlé and were used as replicate samples.
(PDF)

Figure S4 **Heatmap of the OTUs that were both significantly different and had nearly a 2-fold difference in average intensity between inside and outside the brûlé for** ***Pedobacter*** **.** In_1, In_2, In_3 and Out_1, Out_2, Out_3, respectively, were pools from inside and outside the brûlé and were used as replicate samples.
(PDF)

Figure S5 **Heatmap of the OTUs that were both significantly different and had nearly a 2-fold difference in average intensity between inside and outside the brûlé for** ***Flavobacterium*** **.** In_1, In_2, In_3 and Out_1, Out_2, Out_3, respectively, were pools from inside and outside the brûlé and were used as replicate samples.
(EPS)

Figure S6 **Heatmap of the OTUs that were both significantly different and had nearly a 2-fold difference in average intensity between inside and outside the brûlé for** ***Pseudomonas*** **.** In_1, In_2, In_3 and Out_1, Out_2, Out_3, respectively, were pools from inside and outside the brûlé and were used as replicate samples.
(EPS)

Table S1 **Details of each considered truffle-ground and soil chemical parameters for inside (IN) and outside (OUT) the four brûlés sampled.**
(DOC)
