# Supplementary figures and images for: Truffle Brûlés Have an Impact on the Diversity of Soil Bacterial Communities
Source: PLoS One. 2013 Apr 30;8(4):e61945. doi: 10.1371/journal.pone.0061945 (PMC3640031; doi:10.1371/journal.pone.0061945)

Color Key  
and Histogram

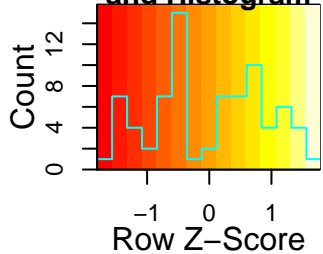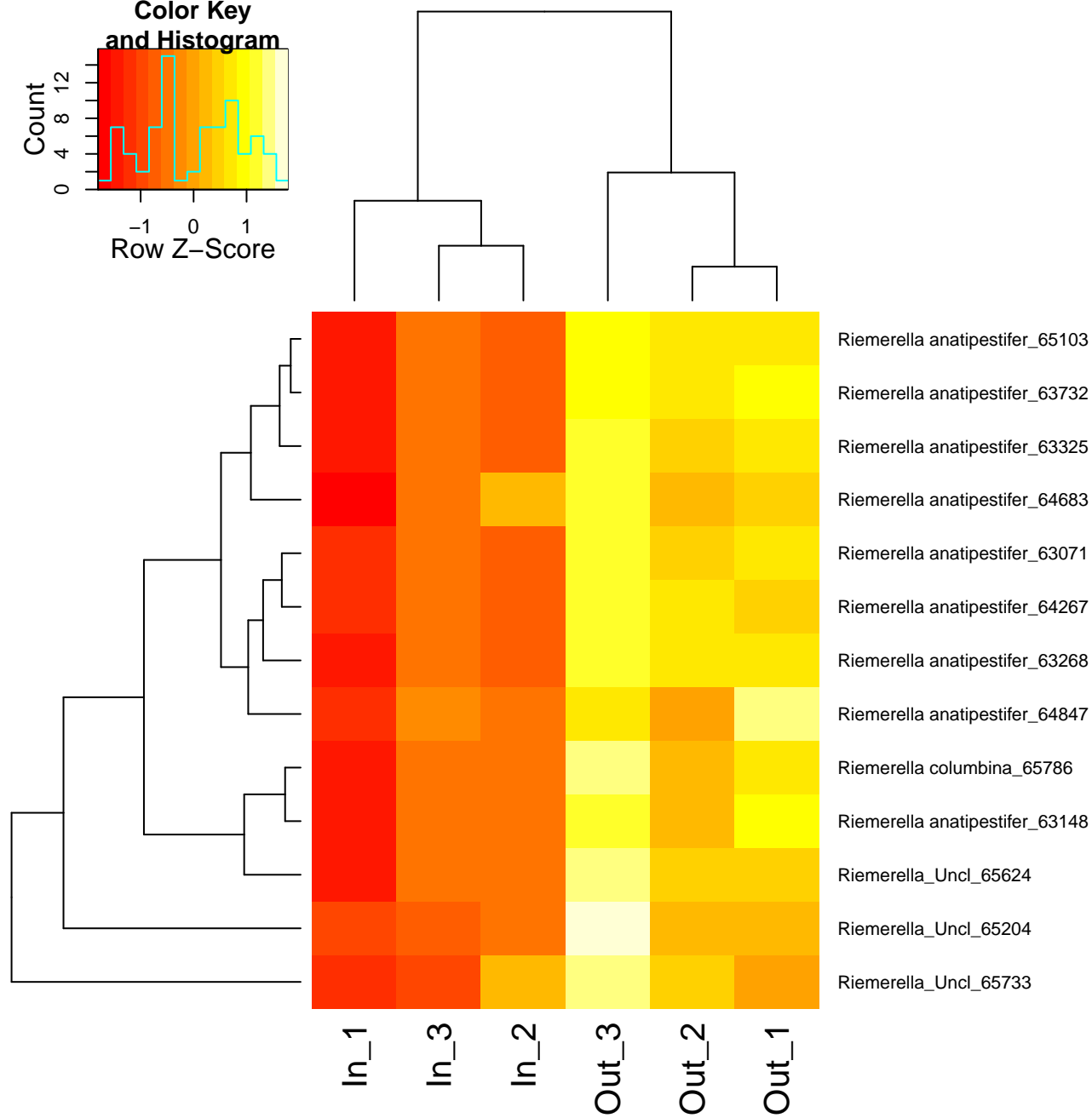

Supplement: Figure S2 — Heatmap of the OTUs that were both significantly different and had nearly a 2-fold difference in average intensity between inside and outside the brûlé for Riemerella . In_1, In_2, In_3 and Out_1, Out_2, Out_3, respectively, were pools from inside and outside the brûlé and were used as replicate samples. (PDF) [file pone.0061945.s002.pdf]

Color Key  
and Histogram

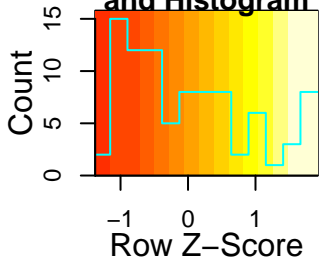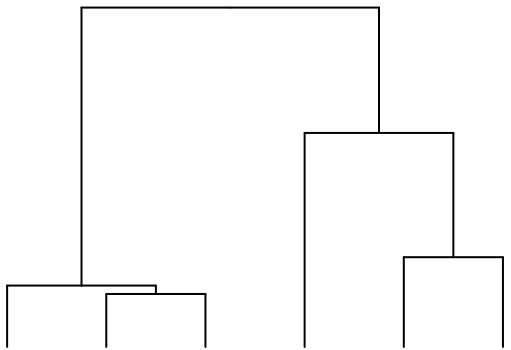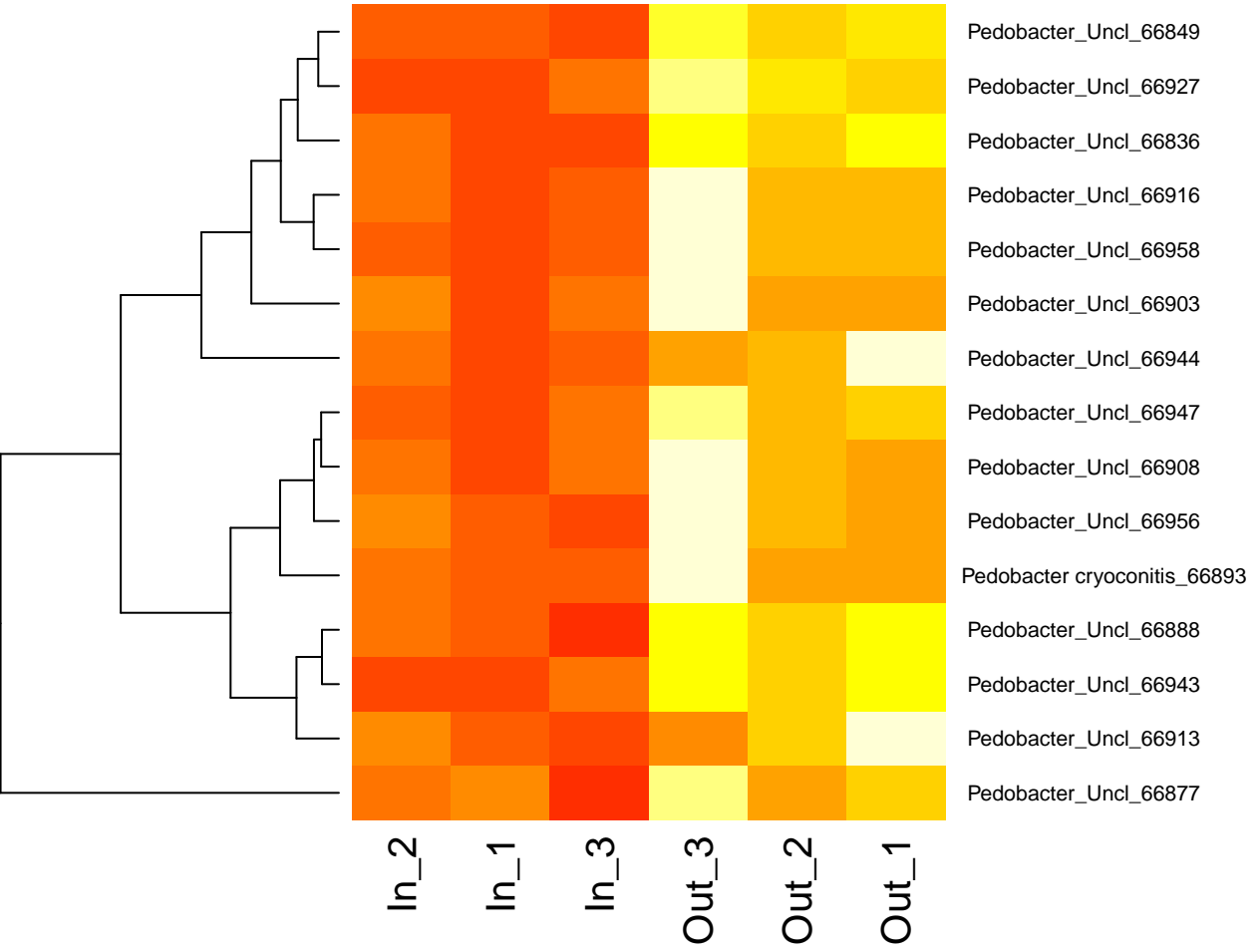

Supplement: Figure S4 — Heatmap of the OTUs that were both significantly different and had nearly a 2-fold difference in average intensity between inside and outside the brûlé for Pedobacter . In_1, In_2, In_3 and Out_1, Out_2, Out_3, respectively, were pools from inside and outside the brûlé and were used as replicate samples. (PDF) [file pone.0061945.s004.pdf]
