# Supplementary material for: Truffle Brûlés Have an Impact on the Diversity of Soil Bacterial Communities
Source: PLoS One. 2013 Apr 30;8(4):e61945. doi: 10.1371/journal.pone.0061945 (PMC3640031; doi:10.1371/journal.pone.0061945)
Supplement: Figure S3 — Heatmap of the OTUs that were both significantly different and had nearly a 2-fold difference in average intensity between inside and outside the brûlé for Chryseobacterium . In_1, In_2, In_3 and Out_1, Out_2, Out_3, respectively, were pools from inside and outside the brûlé and were used as replicate samples. (PDF) [file pone.0061945.s003.pdf]

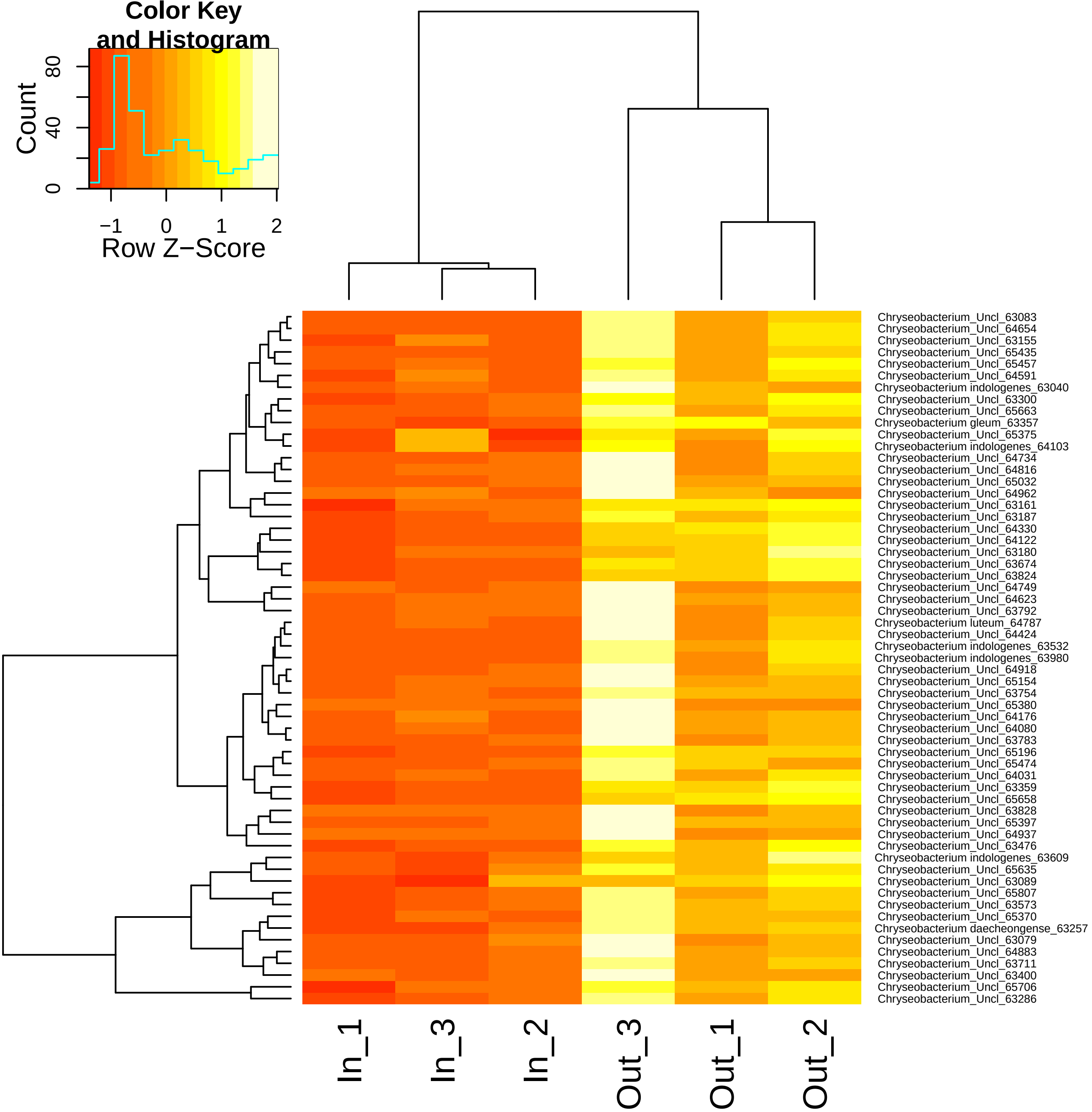
Color Key
and Histogram
Count
0
40
80
−1
0
1
2
Row Z−Score
Chryseobacterium_Uncl_63083
Chryseobacterium_Uncl_64654
Chryseobacterium_Uncl_63155
Chryseobacterium_Uncl_65435
Chryseobacterium_Uncl_65457
Chryseobacterium_Uncl_64591
Chryseobacterium indologenes_63040
Chryseobacterium_Uncl_63300
Chryseobacterium_Uncl_65663
Chryseobacterium gleum_63357
Chryseobacterium_Uncl_65375
Chryseobacterium indologenes_64103
Chryseobacterium_Uncl_64734
Chryseobacterium_Uncl_64816
Chryseobacterium_Uncl_65032
Chryseobacterium_Uncl_64962
Chryseobacterium_Uncl_63161
Chryseobacterium_Uncl_63187
Chryseobacterium_Uncl_64330
Chryseobacterium_Uncl_64122
Chryseobacterium_Uncl_63180
Chryseobacterium_Uncl_63674
Chryseobacterium_Uncl_63824
Chryseobacterium_Uncl_64749
Chryseobacterium_Uncl_64623
Chryseobacterium_Uncl_63792
Chryseobacterium luteum_64787
Chryseobacterium_Uncl_64424
Chryseobacterium indologenes_63532
Chryseobacterium indologenes_63980
Chryseobacterium_Uncl_64918
Chryseobacterium_Uncl_65154
Chryseobacterium_Uncl_63754
Chryseobacterium_Uncl_65380
Chryseobacterium_Uncl_64176
Chryseobacterium_Uncl_64080
Chryseobacterium_Uncl_63783
Chryseobacterium_Uncl_65196
Chryseobacterium_Uncl_65474
Chryseobacterium_Uncl_64031
Chryseobacterium_Uncl_63359
Chryseobacterium_Uncl_65658
Chryseobacterium_Uncl_63828
Chryseobacterium_Uncl_65397
Chryseobacterium_Uncl_64937
Chryseobacterium_Uncl_63476
Chryseobacterium indologenes_63609
Chryseobacterium_Uncl_65635
Chryseobacterium_Uncl_63089
Chryseobacterium_Uncl_65807
Chryseobacterium_Uncl_63573
Chryseobacterium_Uncl_65370
Chryseobacterium daecheongense_63257
Chryseobacterium_Uncl_63079
Chryseobacterium_Uncl_64883
Chryseobacterium_Uncl_63711
Chryseobacterium_Uncl_63400
Chryseobacterium_Uncl_65706
Chryseobacterium_Uncl_63286
In_1
In_3
In_2
Out_3
Out_1
Out_2
