# Supplementary material for: Truffle Brûlés Have an Impact on the Diversity of Soil Bacterial Communities
Source: PLoS One. 2013 Apr 30;8(4):e61945. doi: 10.1371/journal.pone.0061945 (PMC3640031; doi:10.1371/journal.pone.0061945)
Supplement: Table S1 — Details of each considered truffle-ground and soil chemical parameters for inside (IN) and outside (OUT) the four brûlés sampled. (DOC) [file pone.0061945.s007.doc]

| **Truffle ground place** | **Origin** | **Host**  **age** | **pH** | **Texture** | **C/N** | **N (‰)** | **Calcium**  **Carbonate**  **(‰)** | **Organic matter (‰)** | **Total limestone**  **(‰)** | **P (‰)** | **K (‰)** | **Mg (‰)** |
| --- | --- | --- | --- | --- | --- | --- | --- | --- | --- | --- | --- | --- |
| Brûlé 1  La Bigouse, Lalbenque | *Quercus pubescens* plantation | 60  years  old | 7.84 7.76  IN OUT | clay  IN/OUT | 11.5 13.2  IN OUT | 3 3  IN OUT | 11,9 12,2  IN OUT | 59,7 68,3  IN OUT | 40 25  IN OUT | 2,3 2  IN OUT | 0,27 0,31  IN OUT | 0,16 0,19  IN OUT |
| Brûlé 2  La Bigouse, Lalbenque | *Quercus pubescens* plantation | 60 years old | 8.09 7.84  IN OUT | clay clay sandy  loam  IN OUT | 10.8 9.1  IN OUT | 5.2 6.5  IN OUT | 15.7 16.5  IN OUT | 97 102  IN OUT | 150 60  IN OUT | 3.0 3.1  IN OUT | 0.40 0.44  IN OUT | 0,18 0,24  IN OUT |
| Brûlé 3  Le Montat | *Quercus pubescens* plantation | 16 years old | 8.14 8.05  IN OUT | clay sandy loam  IN/OUT | 12.5 13.6  IN OUT | 3.1 3.8  IN OUT | 13.1 13.2  IN OUT | 66.2 88  IN OUT | 400 410  IN OUT | 1.5 1.7  IN OUT | 0.38 0.38  IN OUT | 0,14 0,16  IN OUT |
| Brûlé 4  Escayrac | *Quercus pubescens* plantation | 10 years old | 8.19 8.23  IN OUT | clay sandy clay loam  IN OUT | 12.1 11.5  IN OUT | 2.94 3.0  IN OUT | 14.7 14.6  IN OUT | 61.1 59.7  IN OUT | 200 210  IN OUT | 1.7 1.9  IN OUT | 0.37 0.42  IN OUT | 0,17 0,19  IN OUT |

**Table S1.** Details of each considered truffle-ground and soil chemical parameters for inside (IN) and outside (OUT) the four brûlés sampled.
